# *De novo* characterization of microRNAs in oriental fruit moth *Grapholita molesta* and selection of reference genes for normalization of microRNA expression

**DOI:** 10.1371/journal.pone.0171120

**Published:** 2017-02-03

**Authors:** Xiu Wang, Yisong Li, Jing Zhang, Qingwen Zhang, Xiaoxia Liu, Zhen Li

**Affiliations:** 1 Department of Entomology, China Agricultural University, Beijing, China; 2 Department of Plant Protection, Shihezi University, Shihezi, China; Chinese Academy of Agricultural Sciences, CHINA

## Abstract

MicroRNAs (miRNAs) are a group of endogenous non-coding small RNAs that have critical regulatory functions in almost all known biological processes at the post-transcriptional level in a variety of organisms. The oriental fruit moth *Grapholita molesta* is one of the most serious pests in orchards worldwide and threatens the production of Rosacea fruits. In this study, a *de novo* small RNA library constructed from mixed stages of *G*. *molesta* was sequenced through Illumina sequencing platform and a total of 536 mature miRNAs consisting of 291 conserved and 245 novel miRNAs were identified. Most of the conserved and novel miRNAs were detected with moderate abundance. The miRNAs in the same cluster normally showed correlated expressional profiles. A comparative analysis of the 79 conserved miRNA families within 31 arthropod species indicated that these miRNA families were more conserved among insects and within orders of closer phylogenetic relationships. The KEGG pathway analysis and network prediction of target genes indicated that the complex composed of miRNAs, clock genes and developmental regulation genes may play vital roles to regulate the developmental circadian rhythm of *G*. *molesta*. Furthermore, based on the sRNA library of *G*. *molesta*, suitable reference genes were selected and validated for study of miRNA transcriptional profile in *G*. *molesta* under two biotic and six abiotic experimental conditions. This study systematically documented the miRNA profile in *G*. *molesta*, which could lay a foundation for further understanding of the regulatory roles of miRNAs in the development and metabolism in this pest and might also suggest clues to the development of genetic-based techniques for agricultural pest control.

## Introduction

MicroRNAs (miRNAs) are non-coding small RNAs and are usually 21–24 nucleotide (nt) in lengths [1,2]. Accumulative reports suggest that miRNAs function as important gene expression regulators in almost all known physiological and pathophysiological process in varieties of eukaryotes and viruses [3–5]. Through the specific recognition between the miRNA seed region (nucleotides 2–8 from the 5’ en≈d) and the target sequences of mRNA existing in the 3’, 5’–untranslated region (3’UTR, 5’UTR) or the open reading frame, the finely tuning results of miRNAs normally lead to the degradation [6], transcriptional or translational repression [7,8] or even transcriptional enhancement of the target mRNA [9,10].

Since miRNA lin-4 was firstly discovered in *Caenorhabditis elegans* for regulation of development timing [11], with the development of next generation sequencing platforms, thousands of miRNAs have been identified in human, animals, plants, and viruses [12] and are available on miRBase (http://www.mirbase.org/). Until now, 35828 mature miRNAs have been identified from 220 species, in which 26 species belong to insects (http://www.mirbase.org/). Due to the absence of complete genome sequences, whereas, most of the available sequences of insect miRNAs were determined from model organisms, including 15 species of Diptera (12 *Drosophila*, *Aedes aegypti*, *Anopheles gambiae* and *Culex quingquefasciatus*), 4 species of Hymenoptera (*Apis mellifera* and 3 *Nasonia*), 4 species of Lepidoptera (*Bombyx mori*, *Manduca sexta*, *Heliconius melpomene* and *Plutella xylostella*), 1 Hemiptera species (*Acyrthosiphon pisum*), 1Orthoptera species (*Locusta migratoria*), and 1 Coleoptera species (*Tribolium castaneum*).

miRNA play various regulatory roles in insects, mainly in developmental regulations of germ cell line [13], wing formation [14,15], muscle development [7], neurogenesis [16], apoptosis [17], phenotypic plasticity [18,19], reproduction [18], metamorphosis [20], behavior [21,22], stress resistance [23] and host-pathogen interaction [24,25]. For the multiply regulatory feature, miRNAs are usually expressed in a precisely temporal and spatial pattern [26–29]. Thus, accurate quantifications of miRNA profiles in specific experimental conditions are essential to their functional detection. Up to date, three technologies, microarray [30], bead-based flow cytometry [31], and quantitative real time PCR (qPCR) [32,33] have been developed to study expressional profiles of miRNAs. qPCR, for its sensitivity, flexibility and convenience, has been widely applied to measure miRNA expression levels in diverse organisms. A large number of studies have revealed that appropriate normalizers are critical to the accuracy of gene expression evaluation [34–36].

The oriental fruit moth, *Grapholita molesta* (Busck) (Lepidoptera: Tortricidae) is a worldwide pest of stone and pome fruits in most temperate fruit-growing regions [37] and prefers the new shoots and fruits of host plants within the family Rosacea for boring and internal-feeding [38,39]. *Grapholita molesta* normally has three to five generations per season. The adults migrate from peach orchards to pear or apple orchards. Therefore, outbreaks often occur in the mixed-planting orchards, which result in substantial losses in yields and incomes [40,41]. Nowadays, the main management method is to use insecticides for egg and neonate control. Unfortunately, insecticide resistance has occurred in *G*. *molesta* [42]. An auxiliary technique, pheromone-based mating disruption of adults, is effective but with high management costs [43]. A better understanding about the regulatory mechanisms of the metabolism, development and metamorphosis of *G*. *molesta* would shed light on the development of new techniques for efficient management of this borer pest.

In the present study, on the basis of the Illumina small RNA (sRNA) library sequencing platform, the miRNA transcriptome of *G*. *molesta* was sequenced and performed with systematically *in silico* analysis in reference to the transcriptome of *G*. *molesta* and the genome of *Danaus plexippus*. We also validated the expressional stability of the candidate reference genes and made selection of suitable normalizers under different biotic and abiotic experimental conditions. Our results would provide useful information for revealing the miRNA-involved regulatory mechanism of the development and metabolism in *G*. *molesta* and would also supply a reference for deeper understanding of the expressional profile and regulatory function of miRNA in other non-model insects and agricultural insect pests.

## Materials and methods

### Ethics statement

In this study, the larvae of oriental fruit moth *G*. *molesta* were originally collected in the Institute of Pomology in Liaoning province, China. No permissions were required for the insect collection, as the orchards are experimental plots belonging to the Institute of Pomology in Liaoning province. The “List of Protected Animals in China” excludes insects.

### Insect rearing

*Grapholita molesta* used in this study were reared under laboratory conditions (26 ± 1°C, 70 ± 10%, 15 h L: 9 h D) on fresh apples and artificial diet over four years as our previous study [44]. Different instars of *G*. *molesta* were carefully picked out from rotten apples with soft tweezers. Five larvae of the same instar were reared together in one finger-shaped glass tube (5.5 cm in length ×2.2 cm in diameter) on artificial diet jelly. After pupation, pupae were collected from the cotton-wool tampon on the top of the tubes, and newly-emerged adults were transferred to beakers (1 L in volume) with fresh apples inside for egg laying. Cotton balls soaked with 10% honey solution were provided for nutrient supplement.

### Sample collection and total RNA extraction for sRNA library construction

Healthy samples of *G*. *molesta*, including eggs, the first to fifth instars, prepupae, pupae, female and male adults were collected and snap-frozen in liquid nitrogen. Three hundred eggs, one hundred first and second instars and ten pooled frozen insects of other developmental stages were respectively ground into powder in liquid nitrogen with cooled mortars and pestles. Aliquots of the powdered samples were separately transferred into 1.5 ml RNase-Free Eppendorf tubes. The total RNA samples were isolated with Trizol reagent (Takara, Kyoto, Japan) according to the instruction. The quality and quantity of RNA extracts were then measured with Nanodrop 2000 (Thermo Fisher Scientific, Waltham, MA, USA) and Bioanalyzer 2100 (Agilent, Santa Clara, CA, USA).

### sRNA library construction and sequencing

RNA extracts from each stage of *G*. *molesta* were mixed in an equal amount and then 1 μg of the resulting RNA mix was used for construction of sRNA library using TruSeq Small RNA Sample Prep Kit (Illumina, San Diego, CA, USA). Briefly, sRNAs of 15–30 bp were fractionated on 15% PAGE (polyacrylamide gel electrophoresis); 5’ and 3’ RNA adaptors were then ligated to the recovered sRNA; cDNAs were finally obtained after reverse transcription. After PCR amplification, 6% polyacrylamide TBE (Tris-borate-EDTA) gel purification and cluster formation, the resulting cDNAs were subjected to the single-end sequencing for 50 cycles (1 bp reads per cycle) on an Illumina Hiseq2500 (Illumina, San Diego, CA, USA) in LC-BIO (Hangzhou, China).

### Sequence filter and annotation

The raw reads were subjected to the Illumina pipeline filter (Solexa 0.3) for quality assessment. Clean reads were then obtained through data process with the program ACGT101-miR (LC Sciences, Houston, Texas, USA) after removing adapter dimers, junk reads and common non-coding RNA families (rRNA, tRNA, snRNA, snoRNA). Finally, the unique reads were screened out by eliminating the repeated sRNAs from the clean reads database with single sequence left.

To identify miRNAs, the unique sequence dataset with length of 18–26 bp were mapped in the miRBase (release 21, http://www.mirbase.org/) by BLAST search, and length variations at both 3’ and 5’ ends and one mismatch inside the sequence were allowed in the alignment. The unique reads that mapped to the known miRNAs and/or pre-miRNAs of specific species were determined to be conserved miRNAs. The remaining sequences were further mapped against the transcriptome of *G*. *molesta* (unpublished data) and genome of highly homological lepidopteran *D*. *plexippus* (downloaded from MonarchBase website: http://monarchbase.umassmed.edu). The reads that could map to the transcriptome and/or genome were further subjected to the secondary hairpin prediction using RNAfold software (http://rna.tbi.univie.ac.at/cgi-bin/RNAfold.cgi). The sequences of transcriptome/genome that 20 bp upstream and 60 bp downstream of the mapped sequences and the sequences 60 bp upstream and 20 bp downstream of the mapped sequences were both extracted as candidate precursors for secondary structure prediction. The criteria for secondary structure prediction were: (1) number of nucleotides in one bulge in stem (≤ 12); (2) number of base pairs in the stem region of the predicted hairpin (≥ 16); (3) cut of free energy (kCal/mol ≤ 15); (4) length of hairpin (up and down stems + terminal loop ≥ 50); (5) length of hairpin loop (≤ 20); (6) number of nucleotides in one bulge in mature region (≤ 8); (7) number of biased errors in one bulge in mature region (≤ 4); (8) number of biased bulges in mature region (≤ 2); (9) number of errors in mature region (≤ 7); (10) number of base pairs in the mature region of the predicted hairpin (≥ 12); (11) percent of mature in stem (≥ 80%). The reads with precursors that satisfied all of the above standards and with more than 10 reads account were determined as novel miRNAs. The secondary structure of novel miRNAs were then constructed using srna-workbench V2.5.0 (http://srna-workbench.cmp.uea.ac.uk/downloadspage/) with the precursor sequence and the brief-code of the corresponding secondary structure of each novel miRNA.

### Validation of the Illumina sequencing

To validate the Illumina sequencing, 10 conserved miRNAs that have been reported with relative stable expression in other organisms [45–47] and 10 novel identified miRNAs with high abundance in sRNA library of *G*. *molesta* (**S1 Table**) were selected and their expression level in the sample for construction of sRNA library were checked using qPCR. What’s more, the 20 selected miRNAs were respectively cloned into pMD18-T (Takara) and were further validated with Sanger sequencing by Beijing Genomics Institute (BGI).

### Cluster and conservation analysis

Cluster analysis was conducted among miRNAs at the distance of 3 kb, 5 kb, 10 kb and 50 kb within the genome of *D*. *plexippus*. One cluster was determined when at least two miRNAs could be found in a specific distance range.

The miRNA sequences of arthropods, *C*. *elegans* and *Homo sapiens* have been downloaded from miRBase (release 21). Conservation of miRNA families among *G*. *molesta* and other arthropods was compared through BLASTn analysis. Phylogenetic relationships among 27 insect species belonging to 6 orders in Hexapoda and 4 species in the other three phyla of arthropods have been analyzed. The topology tree was constructed according to previous reports [48,49].

### Target prediction and KEGG enrichment analysis

Putative target genes of miRNAs in *G*. *molesta* were concurrently predicted by TargetScan 50 and miRanda 3.3a through identification of miRNA binding sties on the 3’- UTR sequences of *G*. *molesta* transcriptome. The target genes with context score percentile less than 50 were then eliminated by TargetScan. The ones with max energy greater than -10 were screened out by miRanda. Finally, the target genes simultaneously recommended by the two algorithms were defined as the final prediction.

Functional pathway determination of target genes was performed through the Kyoto Encyclopedia of Genes and Genomes (KEGG) pathway enrichment analysis against the public database (http://www.genome.jp/kegg) [50]. Pathways with *P–*value of fisher’s exact test ≤ 0.05 were determined to be significantly enriched pathways for metabolism or signal transduction. For elucidation of the interactions between miRNAs and target genes in metabolisms and signal transductions, networks of miRNA-KEGG in *G*. *molesta* were further analyzed with miRNA-mRNA target sequence analysis technique and were descripted with Cytoscape 2.8.3.

### Sample preparation and candidate sRNA selection for reference gene selection

Eight experimental conditions had been considered for study on expressional stability of sRNAs in *G*. *molesta*, including two intrinsic biotic conditions (developmental stage and tissue) and six exogenous stress conditions (temperature, photoperiod, starvation, JH injection, dsRNA injection and insecticide).

For samples of different developmental stages, 300 eggs, 200 first instars, 30 second instars, 10 third instars, 10 fourth instars, 7 fifth instars, 10 prepupae, 10 female pupae, 10 male pupae, 10 female adults (3 d old post-emergence) and 10 male adults (3 d old post-emergence) of *G*. *molesta* have been collected respectively as one replicate. For sample preparation of different tissues, 20 fifth instars (2 d old post-molt) and 10 adults (3 d old post-emergence) were dissected under a binocular microscope in 10 mM cold phosphate buffered saline (PBS, pH 7.8) as one replicate; after rinsed in PBS, samples of each tissue, including 9 tissues of larvae (head, cuticle, foregut, midgut, hindgut, Malpighian tubule, fatbody and ventral nerve cord) and 4 tissues of adults (head, thorax, abdomen and leg) were obtained respectively from pooled dissections.

For sample preparation of *G*. *molesta* subjected to stresses of varying temperature, photoperiod and food deprivation, the third instars were treated under nine temperature conditions (4, 26 and 40°C for 2, 12 and 24 h, respectively), eight photoperiod conditions (24 h L: 0 h D, 0 h L: 24 h D, 14 h L: 10 h D and 10 h L: 14 h D for 1 d and 2 d, respectively), and four starvation conditions (starved for 12 h, 24 h, 48 h and 48 h followed by 24 h refeeding). For insect samples pretreated with double strand RNA (dsRNA) and juvenile hormone (JH) injection, the fifth instars were respectively injected with artificially synthesized dsRNA of *met* (encoding putative JH receptor Methoprene-tolerant) and chemical reagent JH III (Sigma-Aldrich, Milwaukee, WI, USA). DsRNA of *egfp* (encoding Enhanced Green Fluorescent Protein) and chemical reagent dimethyl sulphoxide (DMSO, solvent of JH III, Sigma-Aldrich, Milwaukee, WI, USA) were injected as control treatments accordingly. DsRNAs of *met* and *egfp* were artificially prepared with MEGAscript RNAi kit (Ambion) with primer pairs listed in **S2 Table**. For stress induced by insecticide, the third instars were topically applied with LD_50_ of 2.5% β-cypermethrin (1 μL of 1500 times dilution per larva, or 0.625 ng per larvae) calculated using PoloPlus^TM^ software (LeOra software, Berkeley, CA, USA) and were then sampled, respectively, at 12 h, 24 h and 48 h post application. Ten insects pretreated under each exogenous stress condition were collected as one replicate. Three replicates were collected for all of the eight treatments.

All insect samples were snap frozen in liquid nitrogen and stored at -80°C. Total RNA of each sample was then extracted with Trizol reagent as above and checked with Nanodrop 2000 (Thermo Fisher Scientific, Waltham, MA, USA). Subsequently, cDNAs of sRNAs were synthesized after reverse transcription from the qualified RNA with A260/280 value of 1.8–2.3 and A260/230 value ≥ 2.0 using miScript II RT Kit (Qiangen, Dusseldorf, Germany). Universal reverse primer (GAATCGAGCACCAGTTACGC) was used for cDNA synthesis according to the manufacture’s protocol. All of the resulting cDNA samples were stored at −20°C for sRNA expressional analysis.

For reference RNAs selection, 12 sRNAs, including two commonly used normalizer (small nuclear RNA *U6* and small ribosomal RNA *5SrRNA*) for miRNA quantification in mammals and plants [51–54] and 10 miRNAs (bmo-miR-2b-3p_1ss22GC, pxy-mir-6497-p3_1ss7CT, hsa-miR-16-5p, bmo-miR-281-3p_L-2R+2, bmo-miR-279a_R+2, bmo-miR-9a-5p, bmo-miR-998_R+2, bmo-miR-305-5p_R+1, bmo-let-7-5p and mse-miR-92a) which have been used for normalization in previous reports [45–47] and showed abundant expression in the sRNA library of *G*. *molesta* have been selected as candidate reference genes for miRNA profile study of *G*. *molesta*.

### Quantitative PCR analysis

qPCR with high sensitivity and specificity was adopted for validation of sRNA library sequencing and quantification of selected candidate reference genes through measurement of mature sRNA expression.

The forward primers for qPCR amplification of miRNAs were designed according to the specific sequences of miRNAs with some modifications at 5’ ends for adjustment of GC contents (**S1 Table**). The reverse primer for qRT-PCR analysis was the universal primer used for cDNA synthesis above. All primers were synthesized commercially (Sangon Biotechnology, Shanghai, China) and diluted to 10 μM. qPCR was conducted on a Bio-Rad CFX Connect^TM^ Real-Time PCR Detection System (Hercules, CA, US) using miScript SYBR® Green PCR kit (Qiagen). The amplification was performed following the program as: 94°C for 15 min for denaturation, 40 cycles of 95°C for 15 s, 55°C for 30 s, 72°C for 30 s for collection of amplification signal and 70°C for 30 s for reaction termination. The dissociation protocol was also carried out for melting curve analysis on the specific amplification. The amplification efficiency (E%) and correlation coefficient (R^2^) for amplification of each sRNA were calculated according to the standard curve generated from the five 5-fold serial dilution points of cDNAs and their corresponding C_q_ values [35,55].

### Statistical analysis for selection and validation of reference genes

Global mean that was defined as the mean C_q_ value of all expressed sRNAs in a given sample [34] was calculated. The Spearman’s rank correlation coefficient *r*_*s*_ between C_q_ value of each candidate reference gene and the corresponding global mean was then respectively analyzed under each experimental condition [56]. The expressional stabilities of the candidate reference genes were preliminarily ranked according to their *r*_*s*_ values and the sRNA with *r*_*s*_ value closer to 1 was considered more stable.

The expressional stability of candidate sRNAs with *r*_*s*_ value > 0 were further assessed using commonly used software geNorm [57]. The linear scale expression quantity of each candidate gene was calculated by the equation 2^(-ΔCq)^ (according to the handbook of geNorm^TM^ kit with perfect probe, PrimerDesign Ltd.) and loaded in the Excel-based geNorm software. The optimal number of reference genes used under each experimental condition was also recommended by geNorm through pairwise variation analysis.

To validate the selected reference genes, transcriptional level of let-7 in *G*. *molesta* at different developmental stages were detected and compared with different normalizers, including the single one best recommendation hsa16 (NF1), the recommended combination hsa16 + *U6* + bmo2b (NF(1–3)), one least stable miRNA bmo281 (NF8) ranked by geNorm, and single mse92a (NF12) with the lowest *r*_*s*_ value. Relative expression levels of let-7 were calculated according to the ΔΔCq method. The geometric mean calculated from the C_q_ values of the three sRNAs (hsa16, *U6*, bmo2b) were used as normalization factor for NF(1–3). For each specific developmental stage, the significant difference among the expression levels of let-7 calculated according to different normalization factors were statistically analyzed using one-way ANOVA with SPSS Statistics 17.0 (SPSS Inc., Chicago, IL, USA). Specifically, Tukey test was adopted for analysis of gene expression in *G*. *molesta* of fifth instar, male pupa, female pupa, and female adul. Games-Howell test was performed in analysis of gene expression in *G*. *molesta* of first instar, second instar, fourth instar and prepupa. For expressional analysis in *G*. *molesta* of third instar and male adult, Games-Howell test was also applied after square root transformation of the relative gene expression values.

## Results

### Profile of sRNA library

In order to understand the miRNA profile, a sRNA library was constructed with a mixture of RNA extracted from *G*. *molesta* at varying developmental stages. A total of 16,305,575 raw reads were obtained, including 2,219,608 unique sequences. After trimming of the adaptor and junk reads, filtering out the Rfam reads (rRNA, tRNA, snoRNA, snRNA and other Rfam RNA), and removing the repeats, a total number of 5,998,101 unique reads were finally obtained for further analysis (**Table 1**).

**Table 1:** Overview of sRNA sequencing data.

Category	Total reads	Percentage of total reads (%)	Unique sequence	Percentage of unique sequence (%)
**Raw reads**	16,305,575	100.00	2,219,608	100
**3'adaptor & length filter**^***a***^	9,804,065	60.13	1,660,906	74.83
**Junk reads**^***b***^	24,021	0.15	5,511	0.25
**Rfam**^***c***^	477,365	2.93	19,662	0.89
rRNA	328,485	2.01	12,880	0.58
tRNA	62,488	0.38	3,557	0.16
snoRNA	804	0.00	227	0.01
snRNA	2,343	0.01	555	0.02
other Rfam RNA	83,245	0.51	2,443	0.11
**Repeats**	5,190	0.03	456	0.02
**Unique reads**	5,998,101	36.79	533,252	24.02

^*a*^ Length filter: reads with length <18 and >26.

^*b*^ Junk reads: > = 2N, > = 7A, > = 8C, > = 6G, > = 7T, > = 10Dimer, > = 6Trimer, or > = 5Tetramer.

^*c*^ Rfam: collection of many common non-coding RNA families (including rRNA, tRNA, snoRNA, snRNA and other Rfam RNA) except microRNA; http://rfam.janelia.org.

**Fig 1:**
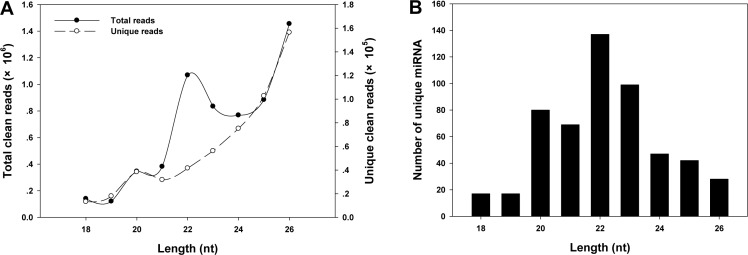
Length distribution of sRNAs in the sRNA library of *G*. *molesta*. **A**, The total number of clean and unique reads of sRNAs in lengths of 18–26 nt; **B**, the number of unique miRNAs ranging from 18–26 nt.

The length of sequenced sRNAs ranged from 18–26 nucleotides (nt). The length of the total clean reads mainly showed two peaks, with the highest one at 26 nt and the other at 22 nt (**Fig 1A**). The number of the unique clean reads increased with the lengths of the reads (**Fig 1A**), and the highest number of miRNAs were found with length of 22 nt (**Fig 1B**).

### Identification of conserved and novel miRNAs

After all of the unique clean reads were subjected to the alignment in the miRBase 21.0, transcriptome of *G*. *molesta* and the genome of *D*. *plexippus*, and were performed with the analysis of the precursors’ hairpin, a total of 536 mature miRNAs were obtained in the sequenced sRNA library of *G*. *molesta* (**S3 Table**). Among the defined miRNAs, 38.06% were mapped to the transcriptome of *G*. *molesta* (2.94% conserved and 97.6% novel) (**S4 Table** and **S1 Fig**), and 27.24% were mapped to the genome of *D*. *plexippus* (67.81% conserved and 32.19% novel) (**S5 Table** and **S1 Fig**). The identified 536 miRNAs were composed of 291 conserved and 245 novel miRNAs which were, respectively, belonged to 206 conserved and 230 novel seed-based miRNA families (**S6 Table**).

**Fig 2:**
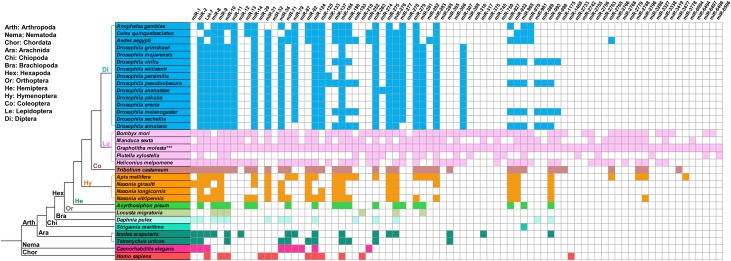
Conservation analysis of 79 miRNA famililes within 33 species. Conservation analysis of the 79 miRNA families identified from the sRNA library of *G*. *molesta* was conducted among 33 species belonging to the three phyla, Arthropoda, Nematoda and Chordata. The miRNA information of the other 32 species was retrieved from the miRBase. Colored box indicated the presence of the conserved miRNA family and the same color indicated species belonging to the same order of insect in Hexapoda, the same class in Arthropoda except for the Hexapoda species, *C*. *elegans* in Nematoda, and *Homo sampiens* in Chordata. The phylogenetic tree was constructed according to previous reports [48,49].

Among the 291 conserved miRNAs, only 40 miRNA showed high expression levels (> 2000 reads) (**Table 2**), and miR-10, miR-8, miR-276, miR-14 and miR-281 were identified as the most abundantly expressed conserved miRNAs in *G*. *molesta*. Majority of the conserved miRNA showed middle (132 miRNAs with 10–1999 reads) to low (119 miRNAs with < 10 reads) expressional levels (**S4**and **S5 Tables**). In order to detect the conservation of miRNAs among *G*. *molesta* and other arthropods, 79 miRNA families identified in the sRNA library of *G*. *molesta* were compared within 31 arthropods, and the phylogenetic relationship among these arthropods were constructed with *C*. *elegans* and *H*. *sapiens* as outgroup taxa (**Fig 2**). miRNAs were relatively conserved in the same order, and the miRNAs were more conserved in orders with closer phylogenetic relationships. Insects appeared having more expressed miRNAs than those in other arthropods, Nematoda and Chordata, and lepidopterans seemed owing the most abundant miRNAs in Hexapoda. In the present study, miR-9 was conserved in all of the insect species, and miR-745 was conserved in Lepidoptera but absent in other species.

**Table 2:** The most abundant conserved miRNAs in the sRNA library of *G*. *molesta*.

miRNA	Sequence	Length	Seed	CG(%)	Free energy	Reads account
bmo-miR-10-5p_L+1	TACCCTGTAGATCCGAATTTGT	22	ACCCTGT	44.20	-37.10	338,584
mse-miR-8	TAATACTGTCAGGTAAAGATGTC	23	AATACTG	50.00	-40.50	330,445
bmo-miR-276-3p	TAGGAACTTCATACCGTGCTCT	22	AGGAACT	44.90	-38	125,388
bmo-miR-14-3p_R+1	TCAGTCTTTTTCTCTCTCCTAT	22	CAGTCTT	40.00	-31.70	55,077
bmo-miR-281-5p_R+1	AAGAGAGCTATCCGTCGACAGTA	23	AGAGAGC	39.80	-39.60	29,116
bmo-miR-11-3p_1ss11AG	CATCACAGTCGGAGTTCTAGCT	22	ATCACAG	56.50	-38.80	27,252
bmo-miR-279d-3p	TGACTAGATTTTCACTTATCCT	22	GACTAGA	39.40	-22.60	26,764
bmo-miR-31-5p_L+1R+1_1ss9AT	AGGCAAGATGTCGGCATAGCTGA	23	GGCAAGA	57.00	-48.20	25,425
bmo-mir-6497-p5	GGAATAAGGATTGGCTCTGAGGAC	24	GAATAAG	64.80	-36.90	24,686
mse-miR-263a_R-1	AATGGCACTGGAAGAATTCACGG	23	ATGGCAC	50.00	-34.10	21,512
bmo-miR-9a-5p	TCTTTGGTTATCTAGCTGTATGA	23	CTTTGGT	40.40	-33.30	21,174
mse-miR-6094	TATTCGAGACCTCTGCTGATCCT	23	ATTCGAG	51.30	-51.40	19,051
bmo-miR-2b-3p_1ss22GC	TATCACAGCCAGCTTTGTTGACT	23	ATCACAG	46.20	-48.30	18,960
bmo-miR-263a-5p_R+4	AATGGCACTGGAAGAATTCACGGGA	25	ATGGCAC	51.50	-40.50	14,803
bmo-miR-71-3p_L-1R+1	TCTCACTACCTTGTCTTTCATG	22	CTCACTA	47.70	-44.50	13,455
mse-miR-277	TAAATGCACTATCTGGTACGACA	23	AAATGCA	51.60	-39.50	8,369
bmo-miR-277-3p	TAAATGCACTATCTGGTACGACA	23	AAATGCA	51.70	-56.50	8,369
bmo-miR-279b-3p_R-1	TGACTAGATCTACACTCATTG	21	GACTAGA	40.50	-47.60	7814.50
bmo-miR-282-5p_L-3R-2	TAGCCTCTCCTTGGCTTTGTCT	22	AGCCTCT	46.00	-37.90	6,909
bmo-miR-750-3p_R+2	CCAGATCTATCTTTCCAGCTCA	22	CAGATCT	48.80	-33	6,646
pxy-miR-274	TTTGTGACCGTCACTAACGGGCA	23	TTGTGAC	43.40	-29.50	6,383
bmo-miR-10-3p	CAAATTCGGTTCTAGAGAGGTTT	23	AAATTCG	44.20	-37.10	5,036
bmo-miR-31-5p_L+1R-2_1ss9AT	AGGCAAGATGTCGGCATAGC	20	GGCAAGA	57.00	-48.20	4,591
bmo-let-7-5p	TGAGGTAGTAGGTTGTATAGT	21	GAGGTAG	52.00	-50.90	4,349
mse-miR-278	TCGGTGGGATCTTCGTCCGTTT	22	CGGTGGG	54.30	-39	4,269
bmo-miR-281-3p_L-2R+2	TGTCATGGAGTTGCTCTCTTTA	22	GTCATGG	39.80	-39.60	4,260
bmo-miR-252-5p	CTAAGTACTAGTGCCGCAGGAG	22	TAAGTAC	37.20	-33.10	4,031
dme-miR-10-5p_L+1_1ss23TA	TACCCTGTAGATCCGAATTTGTA	23	ACCCTGT	47.60	-48.60	3,798
bmo-miR-1175-3p_R-2	TGAGATTCAACTCCTCCAACTT	22	GAGATTC	38.20	-23.50	3,749
mse-mir-8-p5	CATCTTACCGGGCAGCATTAGA	22	ATCTTAC	50.00	-40.50	3,279
bmo-miR-7-5p_R+1	TGGAAGACTAGTGATTTTGTTGTT	24	GGAAGAC	34.70	-24.30	3,037
bmo-mir-6497-p3	GCGTGTCGGGTTTGGACGGGAAG	23	CGTGTCG	64.80	-36.90	2,985
dme-miR-8-3p_R+1	TAATACTGTCAGGTAAAGATGTCC	24	AATACTG	52.50	-51.30	2,914
bmo-miR-133	TTGGTCCCCTTCAACCAGCTGT	22	TGGTCCC	42.70	-39.90	2,828
bmo-miR-305-5p_R+1	ATTGTACTTCATCAGGTGCTCTGG	24	TTGTACT	56.80	-48.30	2,595
bmo-miR-263a-5p_R+5	AATGGCACTGGAAGAATTCACGGGAA	26	ATGGCAC	45.30	-34	2,590
bmo-miR-279c-3p_R+1	TGACTAGATCCATACTCGTCTGC	23	GACTAGA	40.20	-36.90	2469.50
bmo-miR-317-3p_L-2	TGAACACAGCTGGTGGTATC	20	GAACACA	57.00	-41.50	2,318
bmo-miR-2765	TGGTAACTCCACCACCGTTGGC	22	GGTAACT	57.60	-59.80	2,154

bmo: *Bombyx mori*.

mse: *Manduca sexta*.

pxy: *Plutella xylostella*.

dme: *Drosophila melanogaster*.

**Fig 3:**
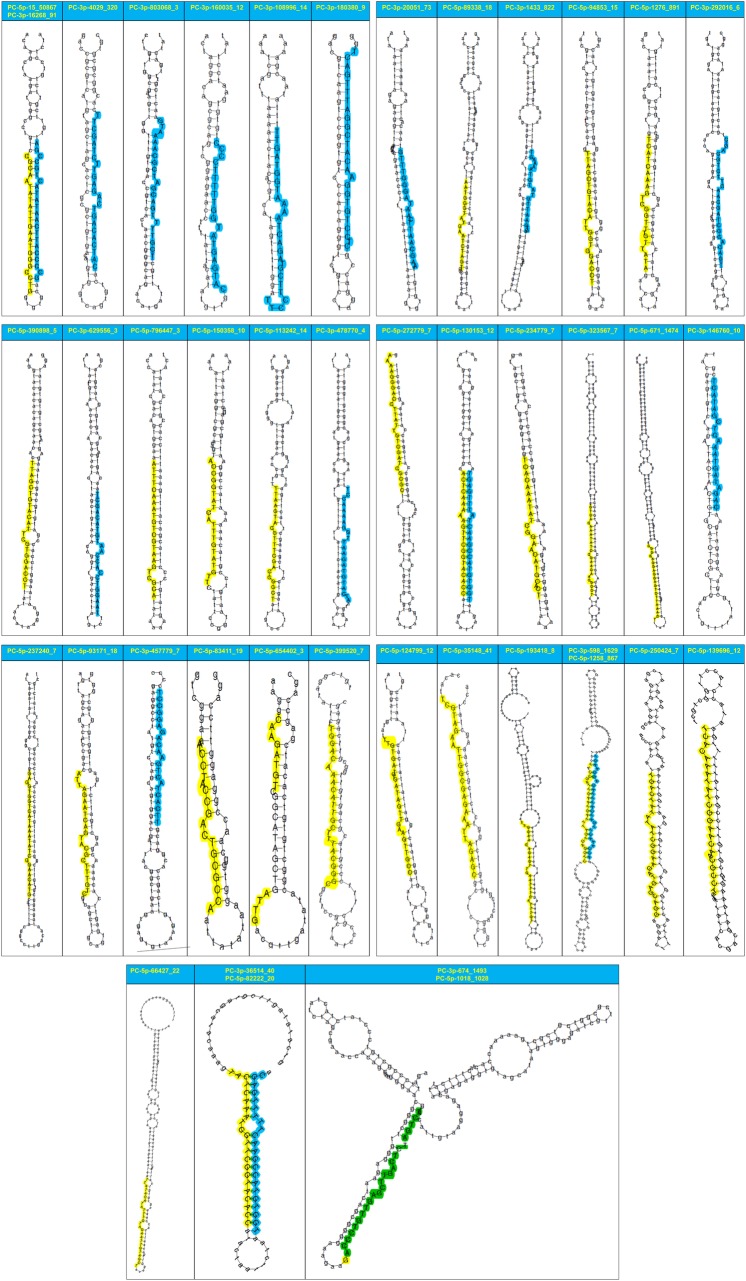
Predicted secondary structures of the 44 novel miRNAs with middle to high abundance in the sRNA library of *G*. *molesta*. The mature miRNAs located in the 5’ arm are shaded in yellow, whereas the mature miRNAs located in the 3’ arm are shaded in blue.

Most (82.04%) of the identified novel miRNAs showed low expressional levels (< 10 reads) (**S4**and **S5 Tables**), 39 (15.92%) of the novel miRNAs showed middle abundance (10–1999 reads) (**Table 3**), only 5 novel miRNAs exhibited with high abundance (> 2000 reads) (**Table 3**). The 5 highly expressed novel miRNAs were gmo-miR-PC-5p-15_50867 (127,819 reads), gmo-miR-PC-3p-598_1629 (17,872 reads), gmo-miR-PC-3p-674_1493 (17,424 reads), gmo-miR-PC-5p-1258_867 (14,307 reads) and gmo-miR-PC-5p-1018_1028 (9,152 reads), all of which accounted about 96.94% of all the identified novel miRNA reads. The secondary hairpin structures of the 44 novel miRNAs with middle to high expressed levels in the sRNA library of *G*. *molesta* were all predicted (**Fig 3**). The 44 miRNAs could be divided into three types: (1) 10 miRNAs derived from either the 5’ or 3’ mature miRNAs of the 5 corresponding precursors, (2) 27 miRNAs only from the 5’ mature miRNAs, and (3) 17 miRNAs only from the 3’ mature miRNAs (**Table 3**and **Fig 3**).

**Table 3:** Novel miRNAs in *G*. *molesta*.

miRNA	Sequence	Length	Seed	CG(%)	Free energy	Reads account
gmo-miR-PC-5p-15_50867	CGCAATATATTGAATGGGCCTG	22	GCAATAT	48.40	-33.10	127,819
gmo-miR-PC-3p-598_1629	ACCAAACTTGATCATTTAGAGGAAGT	26	CCAAACT	49.40	-57.50	17,872
gmo-miR-PC-3p-674_1493	ACCCTGTTGAGCTTGACTCTAGTCT	25	CCCTGTT	50.30	-53.20	17,424
gmo-miR-PC-5p-1258_867	TACCGATTGAATGATTTAGTGAGGTC	26	ACCGATT	49.40	-57.50	14,307
gmo-miR-PC-5p-1018_1028	GACCCTGTTGAGCTTGACTCTAGTCT	26	ACCCTGT	50.30	-53.20	9,152
gmo-miR-PC-5p-671_1474	CGGGAGGAGGAGTGTTAAACT	21	GGGAGGA	65.20	-89.30	1,593
gmo-miR-PC-5p-1276_891	CCATCAAAGTCGGTTTGTTATA	22	CATCAAA	38.00	-30.90	1,050
gmo-miR-PC-3p-1433_822	GTGAATCGTATGTAAAAGT	19	TGAATCG	25.20	-20.20	863
gmo-miR-PC-3p-4029_320	CATCACAGTCGGAGTTCTAGCTT	23	ATCACAG	56.50	-38.80	323
gmo-miR-PC-3p-16268_91	GCCCCATTCAATATACTGCCGA	22	CCCCATT	48.40	-33.10	164
gmo-miR-PC-5p-35148_41	TTGTAGAATTGGCGAGAAATTAGAGC	26	TGTAGAA	52.50	-34.10	159
gmo-miR-PC-3p-36514_40	TGGTGTACCGAACTTTTTGAGT	22	GGTGTAC	37.30	-31.20	121
gmo-miR-PC-3p-20051_73	TAAGCAATTCTATAGGGTTTG	21	AAGCAAT	28.10	-26.70	102
gmo-miR-PC-5p-94853_15	TTAGCTGTACATCGGTGGACCT	22	TAGCTGT	43.60	-55.50	97
gmo-miR-PC-5p-130153_12	ACTCAAAAAGTTCGGTACACC	21	CTCAAAA	38.10	-54.60	72.50
gmo-miR-PC-5p-89338_18	AATGGGTATGCATCTAGTGGC	21	ATGGGTA	40.50	-47.60	46
gmo-miR-PC-5p-82222_20	TACTCAAATAGTTCGGTACACC	22	ACTCAAA	37.30	-31.20	39.50
gmo-miR-PC-5p-83411_19	ATCCTACCGGCTGCGCCA	18	TCCTACC	53.60	-16.90	38
gmo-miR-PC-3p-217431_8	TGGTGTACCGAACTATTTGACT	22	GGTGTAC	38.10	-54.60	35.50
gmo-miR-PC-5p-66427_22	TTATACATATTTGTAGAATTGTAGCT	26	TATACAT	30.20	-41.70	35
gmo-miR-PC-5p-193418_8	TTGGAACAGAGCTGAATTTCATTTGT	26	TGGAACA	29.70	-30.20	26
gmo-miR-PC-5p-93171_18	ATTAGAATCAGTACGCTTTGTC	22	TTAGAAT	46.40	-36.40	24
gmo-miR-PC-5p-323567_7	GTCACAAATATCGGAACAACGCCT	24	TCACAAA	45.50	-66.10	20
gmo-miR-PC-3p-803068_3	NCGTTTTGACGATCGCAAAATG	22	CGTTTTG	44.00	-33.90	20
gmo-miR-PC-5p-113242_14	TTTAAGTACGTTTTGACCGGCT	22	TTAAGTA	44.90	-36.60	18
gmo-miR-PC-3p-146760_10	CAGATGGTAAACTCGATACT	20	AGATGGT	40.70	-35	17
gmo-miR-PC-5p-234779_7	TCACAAATATCTGAACATGCACT	23	CACAAAT	40.70	-44.20	16
gmo-miR-PC-3p-108996_14	TTCCTTCGTAGACTAAAATGGTAGTT	26	TCCTTCG	28.40	-19.70	16
gmo-miR-PC-5p-237240_7	GAGGTGGTCATAAATATCGGAACATC	26	AGGTGGT	45.00	-68.80	16
gmo-miR-PC-5p-139696_12	TCACAAATATCGGAACAACGCCT	23	CACAAAT	40.20	-28.30	15
gmo-miR-PC-5p-272779_7	AAAAGGGACCTTATTGTCGATGGCGC	26	AAAGGGA	43.90	-43.30	15
gmo-miR-PC-5p-399520_7	TGGACAAACATTGCTTTACGGGC	23	GGACAAA	52.40	-22.10	13
gmo-miR-PC-5p-390898_5	TTAGCTGTACATTAGTGGACCT	22	TAGCTGT	44.00	-58.80	13
gmo-miR-PC-3p-180380_9	ATCCTGTGGAACATCGGATTTGACT	25	TCCTGTG	58.00	-40.80	13
gmo-miR-PC-3p-478770_4	ACCATGTAGAATTGGAAAACCT	22	CCATGTA	38.50	-47.30	12
gmo-miR-PC-3p-292016_6	TGACACGCTAGCAACTTCTGCAGC	24	GACACGC	45.80	-42.30	12
gmo-miR-PC-3p-160035_12	CATGAATATGGTTTTTCCCC	20	ATGAATA	41.60	-22.70	12
gmo-miR-PC-5p-124799_12	TTGTGATGTGATAGTTGAAGTTGCC	25	TGTGATG	30.90	-23.50	12
gmo-miR-PC-5p-250424_7	TGACTATATATCGGAAGATGAGCAGG	26	GACTATA	44.00	-24	11
gmo-miR-PC-5p-654402_3	CAAGATGTCGGCATAGCTGATTT	23	AAGATGT	50.80	-25.90	11
gmo-miR-PC-3p-457779_7	TTCACTACTGAACAGAGGCCT	21	TCACTAC	54.40	-40.30	11
gmo-miR-PC-5p-150358_10	TACCGGTATCATTTGTATGTTC	22	ACCGGTA	40.00	-28.20	11
gmo-miR-PC-3p-629556_3	TAAGGTCCACCAATGTACAGCT	22	AAGGTCC	42.60	-46.50	11
gmo-miR-PC-5p-796447_3	ATTTCAAATGTCGTAAGTCGCA	22	TTTCAAA	36.70	-47	10

gmo: *Grapholitha molesta*.

**Fig 4:**
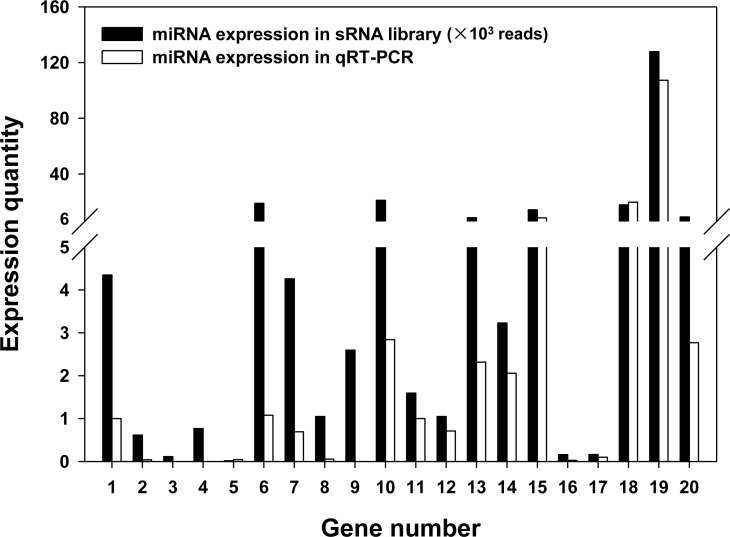
Comparative analysis of qPCR detection with the expressional profiling in sRNA sequencing. The *y*-axis indicates the abundance of miRNAs in the sRNA library and the relative transcriptional level of miRNAs detected using qPCR. The *x*-axis represents the specific assessed miRNAs, as 1: let-7, 2: mse92a, 3: bmo998, 4: pxy-6497-p5, 5: pxy-6497-p3, 6: bmo2b, 7: bmo281, 8: bmo279a, 9: bmo9a, 10: bmo305, 11: PC-5P-671, 12: PC-5P-1276, 13: PC-3P-1433, 14: PC-3P-4029, 15: PC-3P-16268, 16: PC-5P-35148, 17: PC-5P-15, 18: PC-5P-1018, 19: PC-5P-1258, 20: PC-3P-598.

In order to verify the Illumina sequencing, the expressional quantity and sequence accuracy of the 10 conserved and 10 novel miRNA determined in the sRNA library of *G*. *molesta* were validated with the qPCR method and Sanger sequencing. The result of qPCR demonstrated that the abundance of most selected miRNAs showed concordant expressional profiles with the corresponding reads accounts in sRNA library, although bmo9a exhibited very low expressional level in the qPCR test (**Fig 4**). Sequence alignment illustrated that most of the miRNA sequences sequenced by Illumina platform were in accordance with those sequenced by Sanger method. The substitutions at the 5’end produced by the artificially added bases in the forward primer and the differences of one or two bases at the 3’ end resulted from sequencing deviation all have no influence on annotation and target prediction of miRNAs (**S7 Table**). Therefore, Illumina sequencing is a reliable platform for the overall understanding of miRNA profile in *G*. *molesta*.

### Cluster of miRNAs

miRNAs that were transcribed from the same primary miRNA (pri-miRNA) frequently exhibit clustered distribution in the genome and were usually expressed as a polycistronic transcript with co-regulation activity in biological networks. The analysis of the miRNA cluster referred to the genome of *D*. *plexippus* (**Table 4**) revealed that more miRNAs in *G*. *molesta* were linked within 10–50 kb genomic distances, with 28.92% of miRNAs clustered within 50 kb and 26.51% miRNAs closely linked within 10 kb. There were 22.89% and 21.69% miRNAs respectively clustered within 5 kb and 3 kb genomic distance. The mean number of miRNAs per cluster was 3.2, 2.9, 2.7 and 2.8 correspondingly in 50 kb, 10 kb, 5kb and 3 kb genomic distances. At the same genomic distance, a positive and correlated expression relationship has been normally detected among miRNAs within the same cluster ID, or at least two of which appeared with similar expressional profiles. However, the expression patterns (reads account) between distance-neighboring miRNAs of the same cluster varied among different clusters.

**Table 4:** miRNA cluster analysis in *G*. *molesta*.

	Genomic distance			
Cluster ID^*a*^	50k	10k	5k	3k
1	bmo-miR-iab-4-3p_L-3R+1 (10^*b*^)	bmo-miR-iab-4-3p_L-3R+1 (10)	bmo-miR-iab-4-3p_L-3R+1 (10)	bmo-miR-iab-4-3p_L-3R+1 (10)
	bmo-miR-iab-4-5p (59)	bmo-miR-iab-4-5p (59)	bmo-miR-iab-4-5p (59)	bmo-miR-iab-4-5p (59)
	bmo-miR-iab-8_R+2 (20)	bmo-miR-iab-8_R+2 (20)	bmo-miR-iab-8_R+2 (20)	bmo-miR-iab-8_R+2 (20)
2	bmo-miR-34-5p_R+1 (1573)	bmo-miR-34-5p_R+1 (1573)	gmo-miR-PC-3p-1153688_2 (2)	gmo-miR-PC-3p-1153688_2 (2)
	bmo-miR-277-3p (8369)	bmo-miR-277-3p (8369)	mse-mir-278-p5 (36)	mse-mir-278-p5 (36)
	bmo-miR-277-5p_L-1R+1 (18.5)	bmo-miR-277-5p_L-1R+1 (18.5)		
	bmo-miR-317-5p (22)			
3	gmo-miR-PC-3p-1153688_2 (2)	gmo-miR-PC-3p-1153688_2 (2)	gmo-miR-PC-3p-674_1493 (17424)	gmo-miR-PC-3p-674_1493 (17424)
	mse-mir-278-p5 (36)	mse-mir-278-p5 (36)	gmo-miR-PC-5p-1018_1028 (9152)	gmo-miR-PC-5p-1018_1028 (9152)
			bmo-mir-6497-p3 (2985)	bmo-mir-6497-p3 (2985)
			bmo-mir-6497-p5 (24686)	bmo-mir-6497-p5 (24686)
4	gmo-miR-PC-3p-674_1493 (17424)	gmo-miR-PC-3p-674_1493 (17424)	bmo-miR-10-3p (5036)	bmo-miR-10-3p (5036)
	gmo-miR-PC-5p-1018_1028 (9152)	gmo-miR-PC-5p-1018_1028 (9152)	bmo-miR-10-5p_L+1 (338584)	bmo-miR-10-5p_L+1 (338584)
	bmo-mir-6497-p3 (2985)	bmo-mir-6497-p3 (2985)	dme-miR-10-5p_L+1_1ss23TA (3798)	dme-miR-10-5p_L+1_1ss23TA (3798)
	bmo-mir-6497-p5 (24686)	bmo-mir-6497-p5 (24686)		
	gmo-miR-PC-5p-1258_867 (14307)	gmo-miR-PC-5p-1258_867 (14307)		
5	bmo-miR-10-3p (5036)	bmo-miR-10-3p (5036)	bmo-miR-71-3p_L-1R+1 (13455)	gmo-miR-PC-3p-803068_3 (20)
	bmo-miR-10-5p_L+1 (338584)	bmo-miR-10-5p_L+1 (338584)	gmo-miR-PC-5p-1276_891 (1050)	bmo-miR-274-3p_L+1_1ss10GA (1215)
	dme-miR-10-5p_L+1_1ss23TA (3798)	dme-miR-10-5p_L+1_1ss23TA (3798)		pxy-miR-274 (6383)
				pxy-mir-274-p3_1ss10TC (10)
6	bmo-miR-71-3p_L-1R+1 (13455)	bmo-miR-71-3p_L-1R+1 (13455)	gmo-miR-PC-3p-803068_3 (20)	mse-miR-6094 (19)
	gmo-miR-PC-5p-1276_891 (1050)	gmo-miR-PC-5p-1276_891 (1050)	bmo-miR-274-3p_L+1_1ss10GA (1215)	mse-mir-6094-p5_1ss7GA (19)
			pxy-miR-274 (6383)	gmo-miR-PC-5p-201559_9 (9)
			pxy-mir-274-p3_1ss10TC (10)	
7	gmo-miR-PC-3p-1433_822 (863)	gmo-miR-PC-3p-803068_3 (20)	mse-miR-6094 (19)	dme-miR-8-3p_R+1 (2914)
	gmo-miR-PC-3p-803068_3 (20)	bmo-miR-274-3p_L+1_1ss10GA (1215)	mse-mir-6094-p5_1ss7GA (19)	dme-miR-8-5p_R+1 (13)
	bmo-miR-274-3p_L+1_1ss10GA (1215)	pxy-miR-274 (6383)	gmo-miR-PC-5p-201559_9 (9)	mse-mir-8-p5 (3279)
	pxy-miR-274 (6383)	pxy-mir-274-p3_1ss10TC (10)		
	pxy-mir-274-p3_1ss10TC (10)	gmo-miR-PC-5p-113242_14 (19)		
	gmo-miR-PC-5p-113242_14 (19)	bmo-miR-252-5p (4031)		
	bmo-miR-252-5p (4031)			
8	mse-miR-6094 (19)	mse-miR-6094 (19)	dme-miR-8-3p_R+1 (2914)	dme-miR-276a-5p_L-1R-1_1ss22CT (1)
	mse-mir-6094-p5_1ss7GA (19)	mse-mir-6094-p5_1ss7GA (19)	dme-miR-8-5p_R+1 (13)	dme-miR-276b-3p_R+1_1ss10AC (47)
	gmo-miR-PC-5p-201559_9 (9)	gmo-miR-PC-5p-201559_9 (9)	mse-mir-8-p5 (3279)	bmo-miR-276-5p (92)
9	dme-miR-8-3p_R+1 (2914)	dme-miR-8-3p_R+1 (2914)	dme-miR-276a-5p_L-1R-1_1ss22CT (1)	bmo-miR-263a-5p_R+4 (14803)
	dme-miR-8-5p_R+1 (13)	dme-miR-8-5p_R+1 (13)	dme-miR-276b-3p_R+1_1ss10AC (47)	mse-miR-263a_R-1 (21512)
	mse-mir-8-p5 (3279)	mse-mir-8-p5 (3279)	bmo-miR-276-5p (92)	
10	dme-miR-276a-5p_L-1R-1_1ss22CT (1)	dme-miR-276a-5p_L-1R-1_1ss22CT (1)	bmo-miR-263a-5p_R+4 (14803)	tur-miR-1-3p_L-2R+1 (10)
	dme-miR-276b-3p_R+1_1ss10AC (47)	dme-miR-276b-3p_R+1_1ss10AC (47)	mse-miR-263a_R-1 (21512)	bmo-miR-1a-5p_R+1 (38)
	bmo-miR-276-5p (92)	bmo-miR-276-5p (92)		
11	bmo-miR-263a-5p_R+4 (14803)	bmo-miR-263a-5p_R+4 (14803)	tur-miR-1-3p_L-2R+1 (10)	gmo-miR-PC-5p-237240_7 (16)
	mse-miR-263a_R-1 (21512)	mse-miR-263a_R-1 (21512)	bmo-miR-1a-5p_R+1 (38)	gmo-miR-PC-5p-323567_7 (20)
12	bmo-miR-133 (2828)	tur-miR-1-3p_L-2R+1 (10)	gmo-miR-PC-5p-237240_7 (16)	gmo-miR-PC-5p-1071846_2 (5)
	bmo-mir-133-p5 (3)	bmo-miR-1a-5p_R+1 (38)	gmo-miR-PC-5p-323567_7 (20)	gmo-miR-PC-5p-1000352_3 (4)
	tur-miR-1-3p_L-2R+1 (10)			
	bmo-miR-1a-5p_R+1 (38)			
13	gmo-miR-PC-5p-237240_7 (16)	gmo-miR-PC-5p-237240_7 (16)	gmo-miR-PC-5p-1071846_2 (5)	gmo-miR-PC-3p-812930_3 (14.5)
	gmo-miR-PC-5p-323567_7 (20)	gmo-miR-PC-5p-323567_7 (20)	gmo-miR-PC-5p-1000352_3 (4)	gmo-miR-PC-5p-36514_40 (121)
				gmo-miR-PC-5p-130153_12 (72.5)
14	gmo-miR-PC-5p-1071846_2 (5)	gmo-miR-PC-5p-1071846_2 (5)	gmo-miR-PC-3p-812930_3 (14.5)	
	gmo-miR-PC-5p-1000352_3 (4)	gmo-miR-PC-5p-1000352_3 (4)	gmo-miR-PC-5p-36514_40 (121)	
			gmo-miR-PC-5p-130153_12 (72.5)	
15	gmo-miR-PC-3p-812930_3 (14.5)	gmo-miR-PC-3p-812930_3 (14.5)		
	gmo-miR-PC-5p-36514_40 (121)	gmo-miR-PC-5p-36514_40 (121)		
	gmo-miR-PC-5p-130153_12 (72.5)	gmo-miR-PC-5p-130153_12 (72.5)		

^*a*^ miRNAs with same cluster ID number belong to the same one pre-miRNA cluster

^*b*^ The number in the brackets followed the name of miRNA represents the reads account of the corresponding miRNA in the sRNA library of *G*. *molesta*.

PC is short for "putative candidate" and represents novel miRNA.

### KEGG analysis of predicted target genes

**Fig 5:**
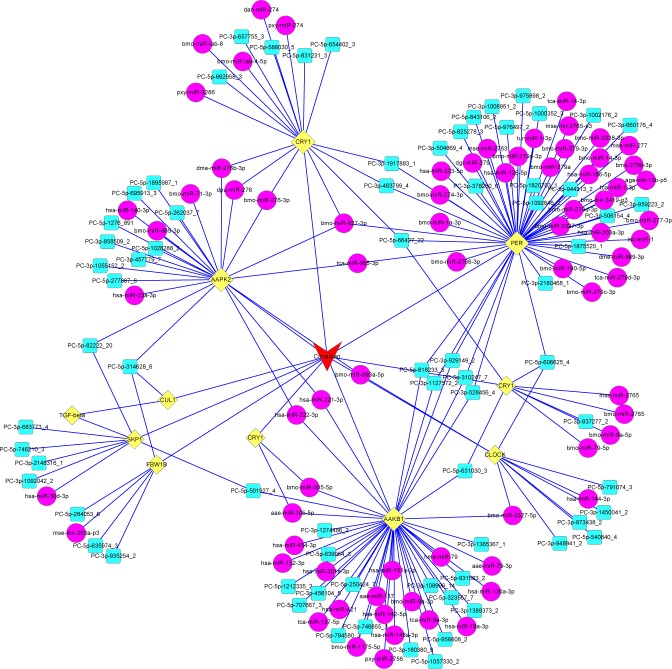
Network prediction of the circadian rhythm pathway in *G*. *molesta*. The miRNAs in magenta and cyan circles respectively represent the conserved and novel miRNAs. Genes in yellow diamonds are clock genes and those involved in growth and development regulation. The regulatory network of miRNAs and their target genes for the circadian rhythm in *G*. *molesta* are linked with blue lines.

KEGG is a database that helps understanding the molecular interaction and reaction networks in cells and organisms (KEGG PATHWAY Database: http://www.kegg.jp/kegg/pathway.html). In this study, the KEGG analysis of predicted target genes of miRNAs was used to increase our understanding about the biological functions of the identified miRNAs in the metabolism and development of *G*. *molesta*. A total of 1,896 target genes of the miRNAs in the *G*. *molesta* were significantly enriched in the KEGG analysis, among which 410 genes were matched to 16 KEGG pathways, including 10 pathways of amino acid metabolism and protein processing (ko00270, ko00330, ko00650, ko00250, ko00280, ko00640, ko00350, ko00410, ko04964 and ko04141), 2 pathways involved in nucleotide repairing (ko03420) and mRNA surveillance (ko03015), 1 pathway of plant-pathogen interaction (ko04626), 2 pathways participating in development regulation (ko04350 and ko04391), and 1 pathway involved in circadian rhythm (ko04710) (**S8 Table**). The further gene network analysis demonstrated that many conserved and novel miRNAs in *G*. *molesta* participated in the circadian rhythm through networks among clock genes (*cry1*, *clock* and *per*) and genes involving in growth and development regulation (*TGF-beta*, *skp1*, *fbw1B*, and *cul1*) (**Fig 5**).

### Expressional stability of candidate reference genes under different experimental conditions

A universal method, mean expression value normalization (global mean), was adopted for preliminarily assess the expressional stabilities of the candidate reference genes. This method demonstrated with better reduction of technical variation and more accurate appreciation of biological change [34]. After calculation and comparison of the Spearman’s rank correlation coefficient *r*_*s*_, eight candidate reference sRNAs (bmo-miR-2b-3p_1ss22GC, pxy-mir-6497-p3_1ss7CT, hsa-miR-16-5p, bmo-miR-281-3p_L-2R+2, *U6* snRNA, bmo-miR-279a_R+2, *5S* rRNA and bmo-miR-9a-5p) were preliminarily identified by their positive correlation with the global mean expressions (**Table 5**), and were further evaluated with geNorm software.

**Table 5:** Information of candidate reference genes used for qRT-PCR.

		Primer information				Correlation to global mean Ct	
Gene name	Symbol	Forward primer sequence(5’-3’)^*a*^	Tm (°C)	E (%)	R^2^	*r*_*s*_^*b*^	*p*^*c*^
bmo-miR-2b-3p_1ss22GC	bmo2b	cccATCACAGCCAGCTTTG	59.7	106.5	0.995	0.952***	0
pxy-mir-6497-p3_1ss7CT	pxy-6497-p3	ccgtGATCTTCCTAGCCGT	59.7	97.3	0.995	0.857**	0.007
hsa-miR-16-5p	hsa16	gaccaggTAGCAGCACGT	59.6	91.4	0.978	0.833**	0.01
bmo-miR-281-3p_L-2R+2	bmo281	gcggcTGTCATGGAGTTG	59.6	102.8	0.993	0.833**	0.01
*U6* snRNA	*U6*	CGCAAGGATGACACGCAA	57.3	126.8	0.991	0.833**	0.01
bmo-miR-279a_R+2	bmo279a	ccgtccgGATCCACACTC	61.9	97.4	0.956	0.69	0.058
*5S* rRNA	*5S*	GCAGTCCACCGAAGTTAAGC	59.8	85.8	0.997	0.407	0.317
bmo-miR-9a-5p	bmo9a	gtcggtcgGTTATCTAGCT	57.6	94.1	0.998	0.347	0.399
bmo-miR-998_R+2	bmo998	gtcagagaGCACCATGGGA	59.7	91.4	0.998	-0.048	0.911
bmo-miR-305-5p_R+1	bmo305	ggcagtGTACTTCATCAGG	57.6	90.7	0.998	-0.071	0.867
bmo-let-7-5p	let-7	cgcgcTGAGGTAGTAGGTTG	61.9	84.6	0.979	-0.143	0.736
mse-miR-92a	mse92a	ccatcccGCACCAGTCC	61.8	102.3	0.985	-0.429	0.289

^*a*^ The upper case letters are nucleotides from corresponding miRNA, and the lower case letters are artificially added for adjustment of GC content in the primer

^*b*^ Spearman’s rank correlation coefficient

^*c*^ Two-tailed tests

*** represents *P*≤0.001

** represents 0.001<*P*≤0.01.

geNorm software ranks the reference gene according to their M values (average expression stability) and provides optimal recommendation of the number of reference genes based on the pairwise variation (V) analysis. In this study, the expressional stabilities of the 8 candidate reference genes were ranked under eight different experimental conditions (**S2 Fig**). The candidate reference, bmo2b, was ranked with high stability under seven experimental conditions, except for temperature treatment; *U6* and bmo9a were all ranked as stable references under four experimental conditions. In combination with the V value (**S3 Fig**), the recommendations of internal normalizers for analysis of miRNA expressional profiles of *G*. *molesta* using qRT-PCR were determined for each of the eight specific experimental conditions. For intrinsic biotic conditions, 3 sRNAs (hsa16, *U6* and bmo2b) used together were recommended for study of different developmental stages. Combined usage of 6 sRNAs (hsa16, bmo2b, *U6*, *5S*, bmo279 and bmo9a) was recommended for miRNA quantification in different tissues (**Table 6**). For different exogenous stresses, it is recommended 2 sRNAs (bmo281 and *U6*) for temperature changes, 3 miRNAs (bmo2b, bmo279 and hsa16) for photoperiod treatment, 2 miRNAs (bmo279 and bmo9a) for starvation, 2 miRNAs (bmo2b and bmo9a) for JH exposure, 2 miRNAs (bmo2b and bmo281) for dsRNA treatment, and 2 sRNAs (bmo281 and *U6*) for insecticide application (**Table 6**).

**Table 6:** Recommended reference genes for expressional qualification of microRNA in *G*. *molesta* under different experimental conditions.

Experimental conditions	Recommended reference genes					
**Intrinsic biotic factors**						
Developmental stage	hsa16	*U6*	bmo2b			
Tissue	hsa16	bmo2b	*U6*	*5S*	bmo279a	bmo9a
**Exogenous impact factors**						
Temperature	bmo281	*U6*				
Photoperiod	bmo2b	bmo279a	hsa16			
Starvation	bmo279a	bmo9a				
JH injection	bmo2b	bmo9a				
dsRNA injection	bmo2b	bmo281				
Insecticide	bmo281	*U6*				

### Validation of reference gene selection

To evaluate the performance of selected reference genes, the expression level of let-7, which participates in development regulation in insects [58,59] and showed dynamic expressional profiles in different developmental stages of *Bombyx mori* [60] was examined in different developmental stages of *G*. *molesta*. Similar expression profiles of let-7 in different developmental stages of *G*. *molesta* were detected when single best recommended reference NF(1) (hsa16) and the optimal recommendation NF(1–3) (hsa16 + *U6* + bmo2b) were used as normalization factors, only with different expression patterns in the second *vs* third instars and fifth instar *vs* male pupa (**Table 7**). However, the use of single least stable reference NF(8) and NF(12), respectively, showed significant down-regulation and up-regulation of let-7 within all tested developmental stages of *G*. *molesta* (**Table 7**).

**Table 7:** Expressional profiles of let-7 in *G*. *molesta* quantified according to different normalizers.

	Normalizers^*§*^			
Developmental stage	NF(1)	NF(1–3)	NF(8)	NF(12)
Egg	1±0.06a^*#*^	1±0.04a	1±0.03a	1±0.06a
1st instar	0.34±0.02c	0.77±0.02b	0d	3.77±0.09a
2nd instar	28.86±5.81c	63.84±3.88b	0.62±0.03c	292.08±19.09a
3rd instar	39.29±3.44b	53.36±1.59b	0.24±0.01c	382.84±28.82a
4th instar	23.74±0.82c	49.83±0.8b	0.22±0.02d	284.74±10.86a
5th instar	93.29±13.37b	150.21±13.85a	0.53±0.07c	78.45±8.25b
Prepupa	50.8±3.33a	54.36±1.01a	0.22±0.02c	19.86±0.17b
Male-pupa	259.19±26.86a	141.35±4.93b	6.61±0.16c	65.62±1.79c
Female-pupa	458.93±25.01a	338.23±7.01b	11.06±0.75d	93.34±4.92c
Male-adult	503.95±19.2b	413.02±29.07b	0.96±0.07c	3325.75±272.5a
Female-adult	120.02±7.43b	106.65±7.58b	0.93±0.04c	239.26±10.66a

^*§*^ Different normalization factors: NF (1), using the single one best reference hsa16 for normalization; NF(1–3), using the recommended combination of the three best reference sRNAs, hsa16, *U6* and bmo2b for normalization; NF (8), using the single least stable reference bmo281 for normalization; NF(12), using single mse92a with the lowest *r*_*s*_ value for normalization

^*#*^ Means within the same line followed by the different letters are significantly different (*P* ≤ 0.05).

## Discussion

The miRNA information of several Lepidopteran insects has been identified with reference to their genomic information [20,61] or genome sequences of the model insect silkworm (*B*. *mori*) [62,63]. In the present study, a pooled sRNA library of *G*. *molesta* was prepared from mixed developmental stages of oriental fruit moth, and was further analyzed in reference to its own transcriptome and the genome of *D*. *plexippus* which was found with the highest similarity to the transcripts of *G*. *molesta* in the phylogenetic conservation analysis (unpublished data). A total of 536 mature miRNAs composed of 291 previously reported and 245 novel ones were finally identified in *G*. *molesta*. In previous studies of Lepidopteran insects, 55 conserved and 202 novel miRNAs were found in *B*. *mori* [61], 163 conserved and 13 novel miRNAs were identified in *M*. *sexta* [20], and 97, 91 and 69 conserved together with 1, 8 and 383 novel ones were respectively identified in the miRNA analyses of *Helicoverpa armigera*, *Spodoptera litura* and *P*. *xylostella* [62,63]. With the cumulative availability of miRNA data and the increase of sequencing depth, more information of mature miRNA would be uncovered and more novel miRNA could be identified in lepidopterans, which would provide useful repertoires for clarification of the modulation complex in insects and other organisms.

Among the 291 conserved miRNAs identified in the sRNA transcriptome of *G*. *molesta*, 40 miRNAs showed highly expressed levels, which probably play critical roles in various regulatory processes in the oriental fruit moth. The miRNAs with the highest account of reads in this study including miR-10, miR-8, miR-281, and miR-263 were also identified as the most abundantly expressed ones in *A*. *gambiae* [64]. miR-10 and miR-8 were found with high abundance in the digestive tract of green bottle fly maggot *Lucilia sericata* and both were considered involved in secretions [65]. A study on *S*. *exigua* demonstrated that miR-10 also participated in developmental regulation since oral feeding using synthetic miRNA mimics of miR-10-1a resulted in suppressed growth and increased mortality [66]. miR-8 showed pleiotropic regulatory functions in insects, including neurogenesis through targeting *atrophin* mRNA or regulating synaptic activity [67,68], homeostasis of innate immunity by altering the expression of antimicrobial peptides [69], and development tuning via responding to ecdysone [70]. miR-281 is another multi-functional miRNA in insects. It is involved in regulation of long chain fatty acid synthesis and cuticle formation in *M*. *sexta* [71], highly expressed in Malpighian tubules and participating in developmental regulation through targeting and suppressing the transcription of ecdysone receptor–B (EcRB) in *B*. *mori* [72], and facilitating the replication of dengue virus (DENV-2) in vector mosquito *Aedes albopictus* by specifically induced in female midgut upon DENV-2 infection [73]. The conserved miR-263 was abundantly expressed in pupae and may participate in temporal regulation during silkworm development [74]. The other two miRNAs, miR-276 and miR-14 that showed reads account at top levels in the sRNA library of *G*. *molesta*, were also found with regulatory effects in the development, reproduction and immunity of insects. miR-276 was noted as one of the miRNAs for identifying reproductive stages of *A*. *mellifera* independent of caste [75] and was found promoting egg-hatching synchrony of locusts through up-regulation of the target, a transcription coactivator gene *brahma* (*brm*) [10]. In studies of *Drosophila*, miR-14 has been considered as an important regulator in the development process, mainly through negative modulation of the Hedgehog signaling [76], positive regulation of the autoregulatory loop of ecdysone signaling [77] and autophagy regulation during salivary gland cell death by targeting 1,4,5-triphospate (IP3) signaling [78]. miR-14 was also found involved in the interactions of insect and virus, e.g. *H*. *armigera* single nucleopolyhedrovirus (HaSNPV) manipulated EcR transcription in *H*. *armigera* through down regulation of miR-14 in host insect [79] and miR-14 in the white-backed planthopper *Sogatella furcifera* may participate in the immune response against the southern rice black-streaked dwarf virus vectored by the planthopper [80].

Analysis about organisms of different kingdoms demonstrated that large fractions of miRNAs share conservation among closely-related species and even species with distant phylogenetic relationships, suggesting evolutionarily conserved regulatory functions across species [81]. In the present study, the 79 miRNA families found in the sRNA library of *G*. *molesta* were detected to be conserved among the Lepidoptera insects, yet most of the miRNA families numbered from miR-1175 to miR-8506 were absent in other species. More miRNAs among the 79 families were detected to be present in Hexapoda species, whereas notably less number of miRNA families were found in species in Brachiopoda, Chiopoda and Arachinida, although they all belong to Arthropods. Let-7 and miR-124 were conserved among Arthropoda, Nematoda and Chordata species in our analysis. Previous analyses suggested that many miRNA families, such as let-7, miR-10, miR-99 and miR-125 were highly conserved among animal species [46], whereas miR-99 was not found in the sRNA library of *G*. *molesta*, while miR-10 and miR-125 were conserved in most species but absent in *C*. *elegans*. Numerous studies have demonstrated that miRNAs showed widely conservative evolution, and thus have been proposed to be used as additional sequence markers in combination of mitochondrial or gnomic DNA for evolutionary modeling and phylogenetic analysis among various taxa [82,83]. Insects have been considered as an excellent model for study about regulatory functions of miRNAs in conserved regulatory pathways between vertebrates and invertebrates [3]. Besides of sequence conservation, miRNAs were also demonstrated with spatial and temporal expression conservation. A comparative study among bilaterian animals suggested that the tissue specificity of ancient microRNAs was highly conserved [84]. A recent study on *Drosophila* discovered that the temporal expression of orthologous microRNAs could be more conserved than their sequences and the hourglass pattern of miRNA expression are highly similar, not only among the miRNAs with highly conserved sequences, but also for rapidly evolving orthologs [85]. Therefore, as an important regulation component at posttranscriptional level between mRNA and gene-encoding protein, miRNA and its expression pattern were considered critical in understanding the evolution of developmental gene expression.

Similar to previous studies in planthopper *L*. *striatellus* [46] and diamondback moth *P*. *xylostella* [63], the abundances of novel miRNAs were also found very low in *G*. *molesta* and the 5 novel miRNAs with the highest abundance may play important roles in the development of *G*. *molesta*. The secondary structure prediction demonstrated that only a small fraction of mature ones were generated from both arms and most of the mature novel miRNAs were produced from either the 3’ or 5’ arms of the hairpins with a slight bias toward 5’ arm usage (5p/3p proportion was around 1.59). The arm usage preference differed in various *Diptera*, such as 5’ arm-bias in *Drosophila* [86], but 3’ arm-bias in *A*. *gambiae* [64]. Studies in mammalians illustrated that selection and usage of preferred arm could be dynamically regulated in a development and tissue specific pattern [87]. The study in *A*. *gambiae* revealed that arm-switching event could happen after blood feeding [64], all of which suggest that miRNAs are expressed under a condition-specific manner and arm switching can significantly enrich the regulatory capacity of miRNA.

As the next-generation sequencing (NGS) method, Illumina sequencing enables the high-throughput and comprehensive understanding about the genome, transcriptome, epigenome or microbiome of a variety of organisms [88–91]. However, Illumina sequencing has been found more prone to produce deviation than Sanger technique, as it is vulnerable to be contaminated in the process of library preparation and accompanied with deviations resulted from base bias and subjective supposition in the bio-information analysis. In the present study, qPCR and Sanger sequencing were adopted for validation of the sRNA library of *G*. *molesta* sequenced by Illumina platform and concordant results between different methods were detected for most of the miRNAs. The divergences between Illumina sequencing and qPCR were mainly due to the limited replication of Illumina analysis, thus, qPCR validation is indispensible for sophisticated measurement about the expressional pattern of specific miRNA of interest. Alignment detected sequence differences happened in some miRNAs between Sanger and Illumina sequencing results. However, most parts of these miRNA sequences were in agreement between the two methods and the seed regions for target prediction were intact after eliminating the sequence deviations at the 5’ end caused by primer modification for adjustment of GC content. Therefore, Illumina sequencing is a reliable platform for the overall understanding of miRNA profile in *G*. *molesta*.

Cluster analysis is an important part for understanding the biological network and evolutionary conservation of miRNA. In this study, the clusters of the conserved and novel miRNAs determined in *G*. *molesta* were analyzed in reference to the genome of *D*. *plexippus*. Fifteen clusters were identified separately at 50 kb, 10 kb, 5kb and 3 kb genomic distances. Most of the miRNAs in the same cluster showed concordant expressional abundance within the same genomic distance. A study showed that expressional levels of clustered miRNAs in mosquito libraries were highly correlated and the mature miRNAs in the same cluster exhibited a strong bias towards the same arm selection [64]. However, the arm selection of the miRNAs in the same cluster showed in a more dynamic manner in this study, with only a small bias towards the 5’ arm selection in the same cluster. Conservation of miRNA clusters among related species would provide evolutionary evidence for functional shift of miRNA in insects. A study found that the cluster organized in mir-2/mir-13/mir-71 was highly conserved in insects [86], which was not detected in *G*. *molesta*. Cluster analysis is highly dependent upon the corresponding genome sequences, hence in future, with the information of genome sequence of *G*. *molesta* and the availability of sequence information in its phylogenetic related species, more useful information of miRNA data would probably be revealed through comparative studies among agricultural pests.

Target prediction and KEGG pathway analysis are important to understand the regulatory networks of miRNAs. In this study, except for the majority pathways in the processing, repairing and surveillance of nucleic acid, amino acid and protein plus one pathway function in the plant-pathogen interaction, three pathways for controlling the growth and development modulation (including two pathways involved in development regulation and one pathway participating in circadian rhythm) were identified in the sRNA library constructed from different developmental stages of *G*. *molesta*. The network prediction of the identified circadian rhythm pathway further revealed the close relationship among a range of miRNAs, clock genes and developmental regulation genes in *G*. *molesta*. A majority of insect developmental processes exhibit circadian rhythm (including growth, ecdysis, metamorphosis, diapause, and eclosion) and developmental circadian rhythm can be profoundly impacted by environmental changes, especially the daily or seasonally thermal and photoperiodic alterations [92]. Correspondingly, most organisms have evolved circadian clock to adjust their development and metamorphism rhythm for coping with environmental changes [93]. Recent studies have detected that the miRNA might be an important regulator of circadian rhythmicity at posttranscriptional level in various taxa of organisms [94,95]. The overall understanding about the network of miRNA and their target genes in the developmental regulation of insects would provide useful information for the monitoring and management of agricultural pest.

Since the discovery about the vital roles of miRNA at post-transcriptional level, precisely expressional profiles of miRNA under specific conditions have been considered as the prerequisite for understanding its regulatory functions. Identical to the quantification of coding genes, using suitable normalizers synchronically processed with tested genes is also considered as an effective way for eliminating the errors from materials and experimental manipulation and realizing accurate evaluation of miRNA profiles [96]. In recent years, numerous studies on selection and validation of reference genes for miRNA quantification have been published being relevant to plants, human, vertebrates and nematode [45,47,97,98], whereas few studies have been reported about insects. In the present study, on the basis of the sRNA library of *G*. *molesta*, one small nuclear RNA *U6*, one small ribosomal RNA *5SrRNA* and ten miRNAs were preliminarily selected as candidate references according to the previous studies [45–47,51–54] and their stabilities under two biotic and six abiotic conditions were further assessed with global mean method and geNorm algorithm. Normally, four statistical methods, geNorm, Normfinder, delta Ct and Bestkeeper are adopted for expressional stability analysis. Based on different algorithms, however, inconsistent outcomes are usually obtained from the four software. Therefore, the comprehensive ranking of expressional stability is hard to determine from parallel analysis with the four algorithms. In the present study, “global mean”, a universal method for analyzing the transcriptional stability of microRNA [34], was adopted for preliminary screening of the candidate reference genes. A final recommendation of suitable normalizers was then clearly acquired with further evaluation by geNorm. In the assessment of reliable internal controls for miRNA expressional quantification in *C*. *elegans*, a genome-wide RNA-seq was conducted for preliminary selection of control miRNAs with minimal variation, and similarly, the final stabilities of candidate reference miRNAs were then ranked by applying the common geNorm logarithm [97]. The comprehensive analysis in the present study detected that intrinsic biotic factors including developmental stages and tissues impacted more on the stability of miRNA expression in *G*. *molesta*, as a greater number of reference genes should be used for normalization in samples of development stages or tissues. Similarly, investigation in the Chinese perch revealed that embryonic developmental stage was an important factor to the variability of miRNA expression [47]. In previous studies of miRNA evaluation, *U6* has always been empirically chosen as an internal control and *U6* has been recommended as a stable reference gene under half of the tested conditions in our study. However, the expressional stabilities of *U6* and other small nuclear RNAs were actually not be acceptable as endogenous controls in many experimental conditions [45,47]. The present and previous studies all illustrated that misusage of an internal control gene would lead to notably distorted results and misunderstanding of gene expressional profiles [99].

In summary, a total of 536 mature miRNAs including 291 conserved and 245 novel miRNAs was identified in *G*. *molesta*. The abundance, conservation and cluster of identified miRNA were analyzed. The KEGG pathway analysis and network prediction of target genes demonstrated that the network composed of miRNAs, clock genes and developmental regulation genes probably play critical roles in the regulation of developmental circadian in *G*. *molesta*. Furthermore, suitable reference genes were selected and validated for study on miRNA expressional profile in *G*. *molesta* under two biotic and six abiotic experimental conditions. The present study provides an overview of miRNA profile in *G*. *molesta* and may serve as a basic reference for evaluation of miRNA abundance in this pest and other insects. Further studies, such as analyzing of the interaction relationship between miRNAs and their targets and deciphering of the regulatory functions and mechanisms of specific miRNAs will shed light on the deeper interpretation of the miRNA-involved post-transcriptional regulation in *G*. *molesta* and might also provide a useful foundation for development of new targets or genetic-based techniques for agricultural pest control in the future.

## Supporting information

S1 Fig Reads frequency of the identified mature miRNAs mapped to different sequence databases. 34.7% was mapped to miRBase, 38.06% was mapped to the transcriptome of *G*. *molesta* and 27.24% were mapped to the genome of *D*. *plexippus*.
(TIF)

S2 Fig Average expression stability and ranking of eight candidate reference genes under eight different experimental conditions calculated using geNorm. The average expression stability (M value) was calculated for each candidate and the sRNA with the lowest M value is considered as the most stably expressed reference gene.
(TIF)

S3 Fig Determination of the optimal number of reference genes for accurate normalization of miRNA transcription under different experimental conditions. Average pairwise variations (V values) were calculated between the normalization factors NFn and NFn+1, and the addition of reference gene is not required when the V value is below 0.15.
(TIF)

S1 Table Information of miRNAs for validation of sRNA library sequencing. (XLSX)

S2 Table Primer information for dsRNA synthesis. (XLSX)

S3 Table Information of all expressed mature miRNAs predicted in the sRNA library constructed with *G*. *molesta* of varying developmental stages. (XLSX)

S4 Table Predicted *G*. *molesta* miRNAs with reference to its own transcriptome data. (XLSX)

S5 Table Predicted *G*. *molesta* miRNAs with reference to the genomic data of *D*. *plexippus*. (XLSX)

S6 Table Seeds-based family distribution of conserved and novel miRNAs identified in *G*. *molesta*. (XLSX)

S7 Table Comparative analysis of the miRNA sequences between Illumina sequencing and Sanger sequencing. (XLSX)

S8 Table KEGG significant enrichment analysis for target genes of miRNAs in *G*. *molesta*. (XLSX)
