# Supplementary figures and images for: De novo characterization of microRNAs in oriental fruit moth Grapholita molesta and selection of reference genes for normalization of microRNA expression
Source: PLoS One. 2017 Feb 3;12(2):e0171120. doi: 10.1371/journal.pone.0171120 (PMC5291412; doi:10.1371/journal.pone.0171120)

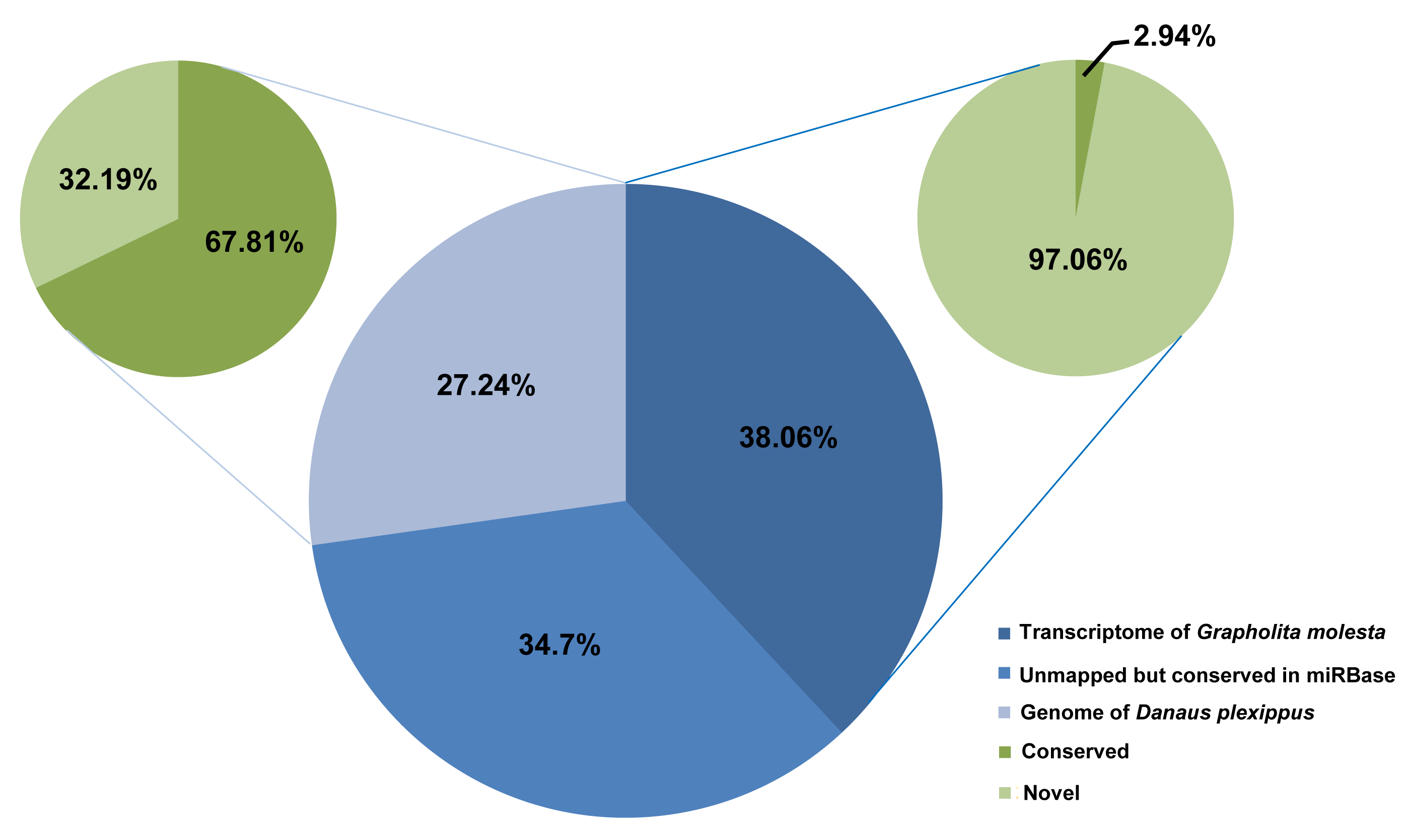

Supplement: S1 Fig — 34.7% was mapped to miRBase, 38.06% was mapped to the transcriptome of G. molesta and 27.24% were mapped to the genome of D. plexippus. (TIF) [file pone.0171120.s001.tif]

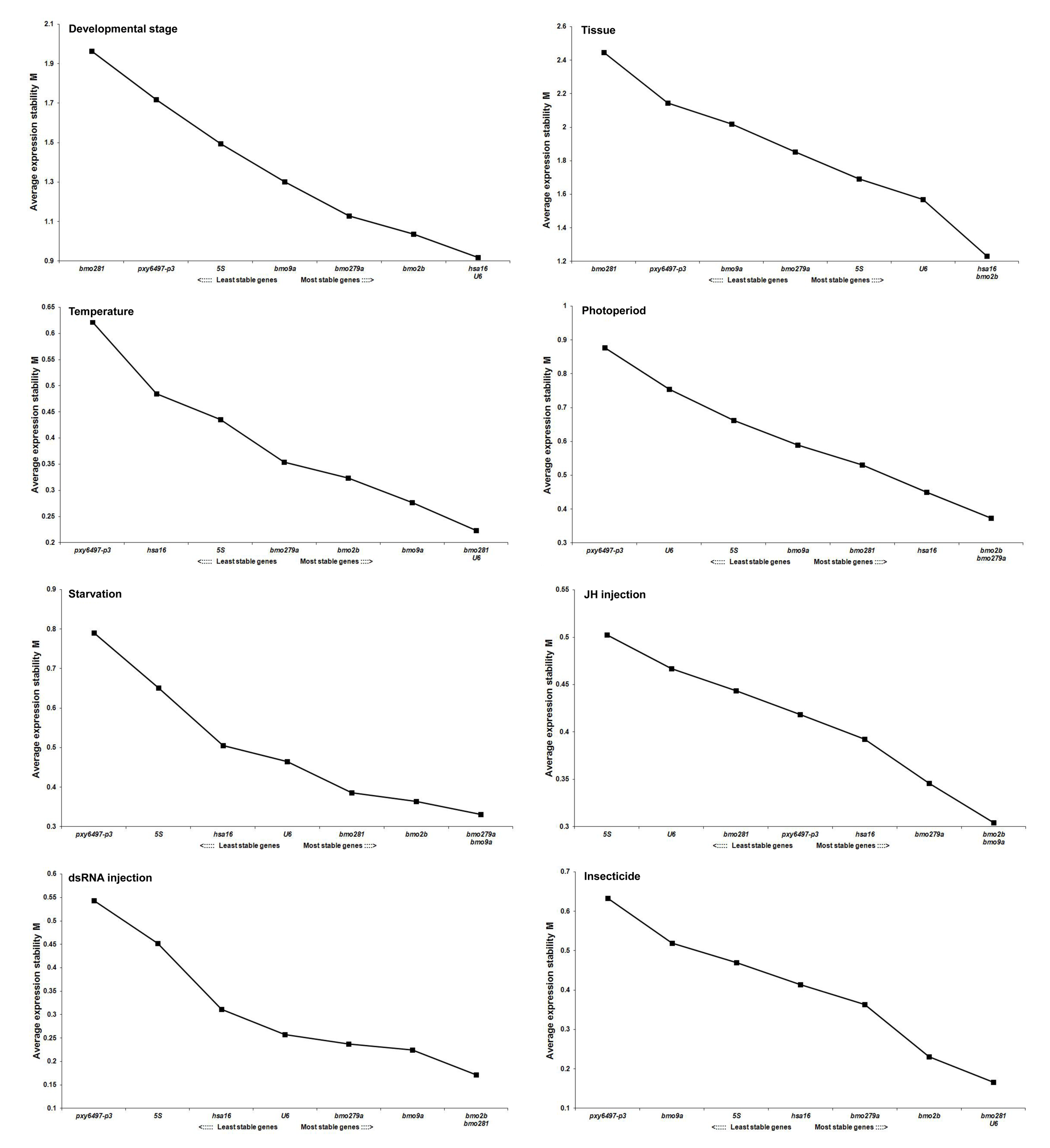

Supplement: S2 Fig — The average expression stability (M value) was calculated for each candidate and the sRNA with the lowest M value is considered as the most stably expressed reference gene. (TIF) [file pone.0171120.s002.tif]

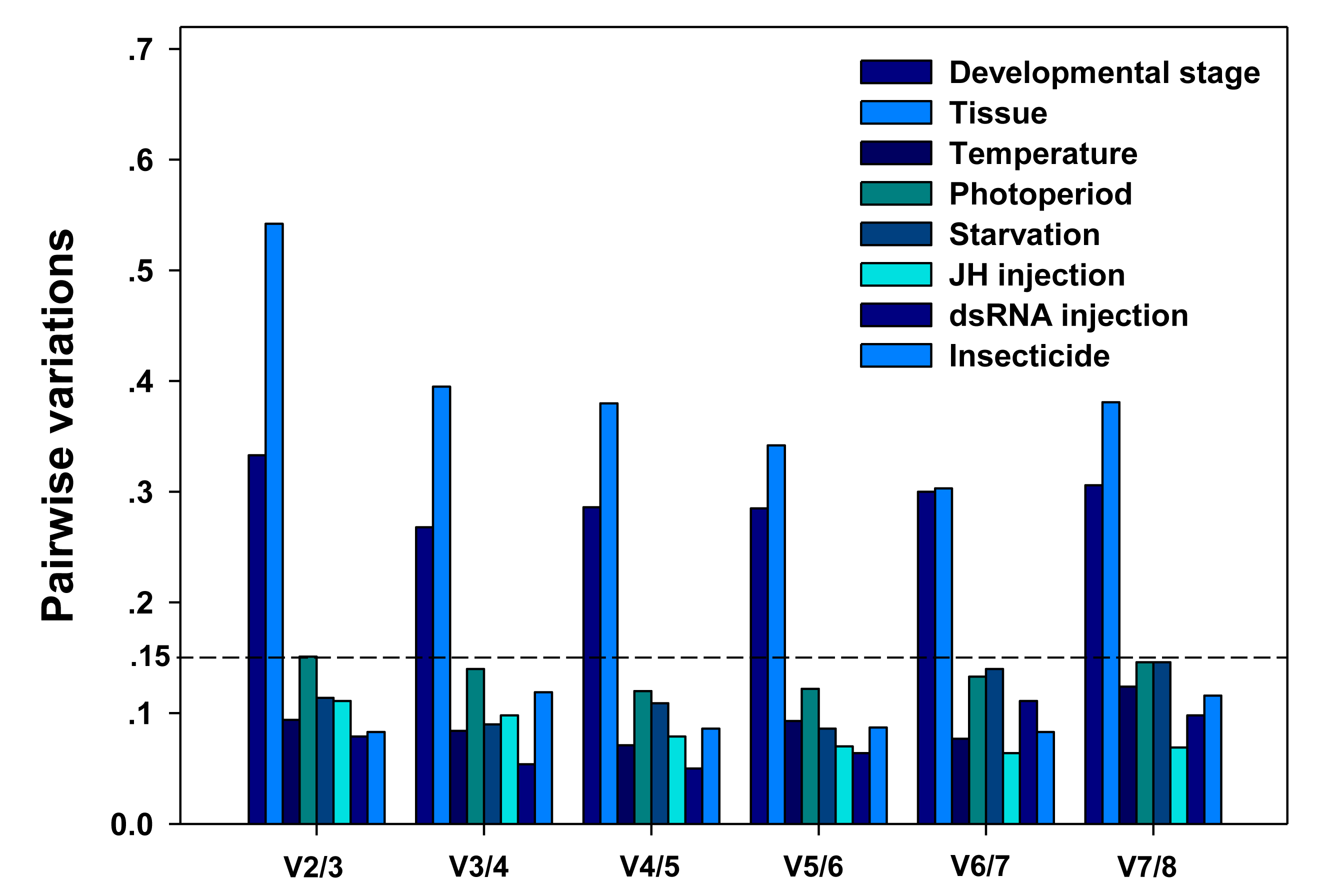

Supplement: S3 Fig — Average pairwise variations (V values) were calculated between the normalization factors NFn and NFn+1, and the addition of reference gene is not required when the V value is below 0.15. (TIF) [file pone.0171120.s003.tif]
